# Does free public transit increase physical activity and independent mobility in children? Study protocol for comparing children’s activity between two Finnish towns with and without free public transit

**DOI:** 10.1186/s12889-020-8385-6

**Published:** 2020-03-17

**Authors:** Arto J. Pesola, Pirjo Hakala, Päivi Berg, Samira Ramezani, Karen Villanueva, Sari Tuuva-Hongisto, Jussi Ronkainen, Tiina E. Laatikainen

**Affiliations:** 1 Active Life Lab, South-Eastern Finland University of Applied Sciences, Raviradantie 22b, 50100 Mikkeli, Finland; 2 Juvenia – Youth Research and Development Centre, South-Eastern Finland University of Applied Sciences, Mikkeli, Finland; 3 Department of Built Environment, Aalto University, Espoo, Finland; 4 Centre for Urban Research, School of Global Urban and Social Studies, RMIT University, Melbourne, Victoria 3000 Australia

**Keywords:** Children’s independent mobility, Physical activity, Sedentary time, Moderate-to-vigorous physical activity, Accelerometer, SoftGIS, PPGIS, Ethnography, Public transport, Bus, Commuting, Built environment, Accessibility

## Abstract

**Background:**

Children’s habitual physical activity, including active travel and catching public transit (walking and cycling to and from destinations), and independent mobility (mobility without an adult) have decreased. Public transit trips are physically active and can provide access to hobbies independent of parents, but there is no device-measured data about children’s total physical activity time following the introduction of free public transit. Our aim is to compare physical activity and independent mobility between children living in two Finnish towns, one with a recently introduced free public transit system, and the other without free public transit.

**Methods:**

The city of Mikkeli has provided free public transit for all comprehensive school children since 2017. Various districts from Mikkeli, and the reference town of Kouvola (towns from South-Eastern Finland with a comparative population size and geographical structure), are selected based on their accessibility and the availability of public transit services. Samples of 10–12-year-old children will be recruited through primary schools. We will compare moderate-to-vigorous physical activity time, sitting time (a thigh-worn Fibion® device) and independent mobility (a participatory mapping method, PPGIS) of children: 1) who live in towns with and without free public transit, 2) who live and go to school in districts with high vs. low perceived and objective access to free public transit, and 3) who report using vs. not using free public transit. In addition, ethnography will be used to get insights on the social and cultural effects of the free public transit on children’s and parent’s everyday life.

**Discussion:**

There is a need for scalable solutions that can increase children’s physical activity independent of their socioeconomic background or place of residence. This project will give information on how a political action to provide free public transit for children is associated with their total physical activity time and independent mobility patterns, therefore providing highly relevant information for political decision-making and for promoting independent physical activity in children.

## Background

Active travel choices, such as walking and cycling to and from places, can form a significant proportion of children’s daily physical activity time [1, 2]. Active travel is a regular and frequent behavior that children can do independently. Despite this potential, children’s independent and active travel and other unorganized forms of physical activities have decreased during the past decades [3–5]. In Finland, the number of schools has decreased by 27% between the years 2000–2011, and potentially due to longer school-travel distance the proportion of primary school children driven to school by their parents has increased from 16 to 20% [6–8]. A similar trend has occurred during leisure time as a consequence to increased number of children participating in organized sports and other hobbies [5]. Children’s independent mobility (CIM) has decreased and replacement of independent active travel choices by private car is one of the main contributors to the total physical activity time decrement in children [8, 9]. Therefore, supporting children’s independent active travel is an important strategy in increasing their daily physical activity time.

Like active travel, public transit use is a regular, frequent and physically active behavior that children can do independently. Yet, public transit use is often ignored as being part of active travel choices. Public transit use has potential to increase total physical activity time through at least two logical mechanisms. Accessing public transit requires walking from the origin to the transit stop, and again to the destination of interest. A median transit-related activity time is 10–20 min in children, therefore forming a significant proportion of public transit users’ daily activity [10, 11]. Despite active travel as a means to increase CIM, there is a drastic drop in active transit in children living outside of a “comfort zone” distance (e.g. 5 km or more) from their places of interest [2, 12, 13]. CIM has decreased particularly in small towns and countryside, where the service network has little by little become sparser [9]. Therefore, public transit can benefit especially children who rarely travel actively, or whose mobility choices are restricted because of their socioeconomic background or distance to their places of interest. The second mechanism how public transit use can increase total physical activity is increasing children’s accessibility of leisure time activities independently [14]. Participation in organized sports and exercise can depend on the accessibility of the specific facilities, car ownership and household income, which undermines children from a low socioeconomic position [15, 16].

Despite public transit use increases physical activity during single journeys, little data exists on the effects of free public transit introduction on children’s whole day physical activity time. Public transit use is positively associated with the whole day physical activity time and the odds of meeting the physical activity recommendations in adults [17, 18]. Children using public transit for school journeys accumulate significantly more device-measured moderate-to-vigorous physical activity time (MVPA) during the whole day compared with both active commuters and those driven to school by their parents [19]. Despite this support for the use of public transit as an important source of the whole day physical activity, it remains unclear if these benefits are amplified by public transit use outside of school hours and following introduction of free public transit policies. Introduction of free bus travel has increased the odds of use in both the young and older people [20, 21]. Moreover, free public transit policy is associated with increased physical activity level and reduced obesity incidence in older adults [21, 22]. Under 18-year-old Londoners were provided free bus travel from 2006, which led to replacing some proportion of short walking trips (< 1 km) with bus travelling [23, 24]. However, there was no evidence of decreased total walking time or total active travel time, whereas the number of car trips and distance traveled by car decreased [23, 24]. Moreover, there was qualitative evidence of an increased independent mobility level and a sense of social inclusion for children [24]. It is noteworthy that in this study children’s physical activity and sedentary time were assessed by surveys, independent mobility was assessed from a subgroup, and the findings are restricted to a highly urbanized area where children already enjoy a higher degree of CIM [9]. To rigorously evaluate the benefits public transit provides for children, it is important to measure children’s independent mobility and objectively assess habitual physical activity during the whole day as a consequence of increased public transit accessibility and/or use in areas where children may have restrictions in their independent mobility, like in small towns and rural areas.

The idea for this study arouse from current policy-debate and the city of Mikkeli’s decision of providing free public transit for all primary and secondary school children for the whole day. Advocates have supported the decision by commenting that the free public transit would decrease the need for extra school transportation services, replace parent’s chauffeur services and increase equality of access to hobbies during leisure time, ultimately leading to increased total physical activity. Others have criticized the decision by pointing out that free public transit would replace children’s active commuting by bike and foot. The aim of this study is to investigate the effects of free public transit provision on the objectively measured physical activity of 10 to 12-year-old children. In addition, we will investigate this effect at different levels of the socio-ecological model, including individual, sociocultural and physical environment levels [25]. We hypothesize that children living in Mikkeli where free public transit is available have more MVPA during school time (including commuting to school) and on leisure time and weekends (improved access to hobbies and a more mobile way of living) compared with their peers living in Kouvola without free public transit. We also hypothesize that children living in Mikkeli have a lower sitting time and a higher degree of CIM due to free public transit. In this paper, we report study design and methods, and for recruitment design purposes, report spatial and public transit service-based accessibility data on Mikkeli and Kouvola regions, Finland.

## Design and methods

The Finnish Basic Education Act guarantees free transportation to all pupils whose school journey is five kilometers or more. In 2017, the city of Mikkeli made a political decision to provide free public transit for all primary and secondary school children for the whole day. FREERIDE is a cross-sectional study comparing physical activity and independent mobility levels in a sample of 10 to 12-year-old children living in Mikkeli as compared to children living in Kouvola, a reference town without free public transit. Mikkeli (54,000 residents) and Kouvola (83,000 residents) are small towns located in South-Eastern Finland and have a similar climate, geographical structure and possibilities for active transit, including an active bus network for local traffic. Children are being recruited through primary schools located in neighbourhoods that are paired between the towns based on objectively analysed public transit accessibility.

### Primary outcome

The primary outcome is device-measured MVPA (minutes per day) assessed as a daily average of a seven-day measurement period and compared between children living in Mikkeli and Kouvola. Minimum difference of interest (MDI) is 15 min/day of MVPA. We expect a 6% (ρ = 0.06) school-level intraclass-correlation and assume average *n* per cluster of 20 (design effect = 2.1 estimated with formula 1 + (n-1)ρ). A sample size of 200 for each town spread across 10 clusters is required to have ≥80% power to detect MDI at 5% alpha error level (two-tailed significance).

### Secondary outcomes

Our secondary outcomes are device-measured sitting time and CIM compared between children living in Mikkeli and Kouvola. CIM will be assessed with a Public Participatory GIS (PPGIS) questionnaires, which both the participating children and their parents will answer (*child PPGIS questionnaire* and *parent PPGIS questionnaire*).

### Mechanisms

In addition to the town-level comparisons, we will compare MVPA, sitting time and CIM between children having a subjectively and objectively better versus worse access of public transit (neighbourhood level effects), as well as between children using versus not using public transit (individual level effects). We expect to see benefits from public transit use on MVPA and sitting time during school commuting [19], commuting to leisure destinations, and as a consequence of better accessibility of hobbies during weekdays and weekends [15] (domain level effects). Moreover, we will investigate the degree to which travel cost (free vs. non-free) influences children’s travel mode choice (or CIM) and their MVPA while considering the effect of other latent (e.g. children’s perceptions of safety and security, children’s perception of transit accessibility, parent’s perceptions of safety and security, parents’ perceptions of transit accessibility and of degree of environmental accountability) and objective (e.g. objective accessibility measures) factors. We will also conduct a qualitative ethnographic study to investigate social and cultural interpretations of free public transit from the perspective of children and their families. The ethnographic study will be conducted with 6–12 families living in Mikkeli and Kouvola areas differing in their public transit accessibility (1–2 families from clusters 1–6, 1–2 families from clusters 7–8 and 1–2 families from clusters 9–10 per town).

## Neighborhood and school selection

In order to create comparable school pairs for the study, we conducted analysis for school pairings. Thus, multiple public transit accessibility measures for each school were assessed. This was done after determining the sample size but prior to contacting schools, participant recruitment and data collection.

Most studies on public transit accessibility focus on assessing proximity to public transit stop or number of available public transit stops in a given area [26–29]. However, to ensure that the public transit accessibility measures in this study are sensitive enough to create school pairings, additional accessibility measures for each school were calculated. First, each school was categorized based on their allocated urban zone. This was done by using the YKR Urban Zones dataset provided by the Finnish Environment Institute (SYKE). YKR Urban Zones data is a 250 m × 250 m grid-based dataset in which all city regions in Finland are divided into different zones based on three main criteria: distance to the city center, public transit frequency and walking distance to public transit stops. Furthermore, these criteria are calculated for each YKR grid cell which is then assigned a value indicating if it belongs to walking, public transit or car zone [30]. For our study, each school was assigned with an YKR urban zone based on the YKR grid they are within.

In addition to determining which YKR zones each school belongs to, a 1000-m network buffer service area for each school was created and public transit accessibility within each of these service areas was assessed. Buffers of 400 to 600 m are commonly used measures in public transit and urban planning field to identify the area from which most transit users access the system by foot [28, 31]. For this study the network distance for each school was increased to 1000 m, as residents’ willingness to walk to public transit stop can be much higher [31, 32], especially in lower-density areas [33]. Moreover, some of the schools did not have any public transit stops within an 800-m network distance buffer which confirmed the need for increased buffer size.

Public transit accessibility was assessed within the 1000-m school network buffer. The total number of public transit stops and public transit stop density value (number of stops / service area m2) for each school was calculated. In addition, we also created measures of public transit service level for each school using transit timetable data from both cities [28]. For each public transit stop we assigned a frequency value representing the number of trips through the stop during 1 day. In addition, we calculated the street network distance from each school to the city center (main railway station). Finally, a school public transit accessibility dataset was created including all schools from both cities with measures indicating which city the school belongs to, the YKR Urban Zone of the school, frequency and density of public transit stops within 1000 m network buffer around the school, total and average number of trips per day and per hour within 1000 m network buffer around the school and total and average number of trips during commuting and non-commuting hours within the 1000 m network buffer. Finally, we created 10 school pairs to reach a sample size of 200 children for each town spread across 10 clusters (schools) (Table 1).

**Table 1:** Objective public transit (PT) accessibility measures of each school in Mikkeli and Kouvola and school pairs of the study

Pair nr.	City	School id	YKR zone^a^	PT stop density (PT stops per school network area)	PT trips per day	Average PT trips per day	Daily PT trips per school network area	PT trips per hour	Hourly PT trips per school network area	PT trips 07–10 am	Average PT trips 07–10 am	PT trips on weekdays	Average PT trips per weekdays
1	Mikkeli	1007	2	15	910	51	555	38	23	181	10	500	28
	Kouvola	2011		10	344	31	268	14	11	65	6	172	16
2	Mikkeli	1010	41	18	1646	57	998	69	42	281	10	784	27
	Kouvola	2023		14	1190	70	734	50	31	253	15	607	36
3	Mikkeli	1012	5	10	674	48	478	28	20	80	6	300	21
	Kouvola	2029		9	544	49	429	23	18	110	10	290	26
4	Mikkeli	1013	5	8	400	40	313	17	13	51	5	163	16
	Kouvola	2007		6	418	60	331	17	14	99	14	264	38
5	Mikkeli	1016	5	7	52	9	55	2	2	23	4	52	9
	Kouvola	2000		4	107	27	75	4	3	24	6	64	16
6	Mikkeli	1006	5	12	249	16	182	10	8	50	3	138	9
	Kouvola	2006		6	93	16	98	4	4	12	2	93	16
7	Mikkeli	1003	5	8	689	57	420	29	18	119	10	356	30
	Kouvola	2026		5	364	46	230	15	10	66	8	158	20
8	Mikkeli	1018	0	12	172	11	123	7	5	52	3	117	8
	Kouvola	2022		7	92	9	65	4	3	27	3	92	9
9	Mikkeli	1005	0	9	117	17	78	5	3	23	3	100	14
	Kouvola	2033		7	169	19	127	7	5	45	5	121	13
10	Mikkeli	1022	0	8	221	18	149	9	6	71	6	162	14
	Kouvola	2031		8	314	31	252	13	11	90	9	174	17

^a^ 2 = walking zone, 41 = intensive public transit zone, 5 = car zone, 0 = out of urban zones

## School and participant recruitment

**Fig. 1:**
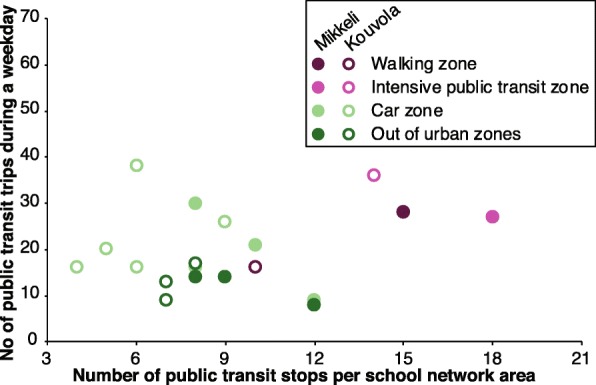
Average number of public transit trips during weekdays as a function of public transit stop number across the Mikkeli and Kouvola YKR zones

Based on the objectively assessed public transport accessibility (Table 1 and Fig. 1) participating children will be recruited through paired primary schools across Mikkeli and Kouvola. We have received permission to contact schools from the head of local education and culture department of both towns. The recruitment of schools will be done in three stages: 1) contacting the principals and 4th and 5th grade teachers of schools 2) a short oral presentation about study protocol given by researcher 3) a participation agreement from the principals of the schools.

We will recruit and measure the paired schools from the Mikkeli and Kouvola simultaneously (e.g. measurements are started in Mikkeli on Monday and in Kouvola on Tuesday). We aim to measure one 4th grade and one 5th grade class per school, with the aim to recruit a minimum of 20 children per school. A researcher will send via email or deliver personally an information letter and an informed consent to the teachers, who will deliver them to parents/guardians via children. Children’s participation in the data collection will be voluntary and they are required to return the consent form signed by their parents before taking part in the measurements. Parents will declare their participation in the same form.

The recruitment is scheduled to start in February 2020 and the first measurements in March 2020. The measurements will be conducted during snow-free time.

## Measurement protocol

The measurement protocol per school requires two visits by the researcher. The researcher will send informed consent forms to the school before the first visit. The children are asked to return the written informed consent for the first researcher visit. At the first visit, the children will complete the *child PPGIS questionnaire* in the computer class of the school under the researcher supervision. Because of the questionnaire nature, it can be integrated into Environmental Education or Biology lessons. The lesson will be agreed with the responsible teacher beforehand. The researcher will measure height and weight, provide the accelerometer and give instructions for the physical activity measurements and completing the *PPGIS mobility log* on one child at a time during the first visit. The children will be asked to continue their normal daily life as they usually do, while filling in the log and wearing the accelerometer for 8 days. They will be asked to return the accelerometer to the teacher after the measurement period and the researcher collects the devices at the second visit. A website link to the *parent PPGIS questionnaire* will be sent to the parents/guardians via e-mail after their children’s 8 days measurement period. The parents’ email address will be obtained from their informed consent.

## Measurements

### Accelerometer

Children will wear a Fibion® device (20 g, L = 30 mm, W = 32 mm, T = 10 mm; Fibion Inc., Jyväskylä, Finland) for 8 days, 24 h per day. At day one, the measurement will be started during the school day and the following full 7 days will be utilized for analysis. The Fibion® device will be attached on the participant’s right thigh. The device will be positioned vertically at the centerline and horizontally at the upper third level on the anterior side of the thigh and secured in a waterproof covering with medical adhesive tape. The Fibion® device measures raw acceleration on three axes with an internal sampling rate of 12.5 Hz. The Fibion® device has no buttons or display and can operate for around 30 days on full charge condition. Fibion® is valid in detecting sitting and continuous uninterrupted sitting periods against direct observation, and light physical activity, MVPA and total energy expenditure against indirect calorimetry [21]. Moreover, Fibion® gives estimates of activity types including standing (mean difference 17,2 min/12 h day, limits of agreement (LoA) -12,9 to 47,3 min), walking (mean difference − 17,3 min/12 h day, LoA − 47,4 to 12,8 min) and cycling duration (mean difference − 6,8 min/12 h day, LoA − 18,7 to 5,0 min) [34]. Fibion® has been used to monitor children’s daily activity previously [35].

### PPGIS

PPGIS (also referred to as SoftGIS) is an online method developed to integrate the human behavior, preferences, experiences and ideas to actual physical settings and to the datasets of GIS system [36, 37]. SoftGIS methodology relies on collecting, analyzing, and delivering soft, geocoded knowledge produced by respondents. PPGIS methods enable a unique collection of large data sets producing scientifically high quality spatial research knowledge and findings, which have been shown to be easy to translate to practical knowledge and proposals for action [38]. In this study, children will keep a *PPGIS mobility log* on their mobile phone or desktop computer web browser, where they map their school, hobbies and other destinations, time spent at and travel mode to destinations during the seven-day accelerometer measurement period. Children will also report sleep times (light out to waking up) and any accelerometer removal periods or other abnormal occasions that may affect the measurement. With the mapped elements a spatial activity space for each child can be delineated [39]. In addition to spatial elements, the *child PPGIS questionnaire* filled in school includes questions of children’s independent mobility [40], self-assessed PA (Short-form international physical activity questionnaire, IPAQ), subjective accessibility of (free) public transport [41], children’s usage of (free) public transport, perceived health and parental support for children’s physical activity [42]. The *parent PPGIS questionnaire* includes questions regarding their children’s mobility licenses [40] as well as self-assessed PA (IPAQ), subjective accessibility of public transport [41], own usage of public transportation and private car and finally their environmental attitudes and socioeconomic status of family.

The PPGIS questionnaire will be supplemented with actual GPS recordings (Sensedoc™ 2.0; MAX-M8 Global Navigation Satellite System receiver from u-blox, 2 s epoch, Tri-axial accelerometer, 50 Hz) with a minimum of 30 children per town. The GPS data is collected to compare and validate how the PPGIS data about children’s’ activity spaces match with the GPS tracks and accelerometer data collected from each participating child. The children participating in the testing phase will wear GPS devices for two consecutive days and answer the PPGIS survey post-GPS measurement. GPS devices with built in accelerometers are big and expensive devices. Moreover, GPS data collection and analysis is highly resource demanding. Thus, as part of this research project we want to study the potentials of combining resource modest PPGIS surveys and easy-to-wear accelerometer devices for comprehensive objective and subjective spatially referenced physical activity data collection.

### Ethnography

The aim of the ethnographic study is to understand the social and cultural effects and mechanisms of the free bus experiments in children’s and parent’s everyday life. The everyday mobility shapes and structures the family life and the children’s individual mobility is at the core of the family-relations: it is the arena of intertwining restrictions, fears, attitudes and recommendations. The ethnographic study provides understandings and a thick description [43] of the broader everyday mobility patterns [44], the social and cultural contexts were the free public transit intervenes [45, 46].

Ethnography refers to the description of cultural systems or an aspect of culture based on fieldwork in which the investigator is immersed in the ongoing everyday activities of the designated community for the purpose of describing the social context, relationships and processes relevant to the topic. At the core is the fieldwork, which includes field note, in-depth interviews, guided-tour-interviews and observations.

## Outcomes

*MVPA, light activity, sitting, standing, walking and cycling time* will be assessed with the Fibion® device as a daily average of a seven-day measurement period, and separately for travel time, school time, non-school time and hobbies. Fibion® uses device orientation and impact data to estimate these postures and activity classes. Data from the Fibion® devices will be uploaded from the device to the manufacturer’s web-browser-based online service and the participants’ weight, height, age, and sex will be submitted to the service. The service analyses the data and provides day-by-day and minute-by-minute results for each activity class duration (in seconds) and energy expenditure (in METs) in a CSV format. The Fibion® service automatically analyses non-wear time as > 30 min periods when the device remains still. MVPA is analyzed by summing the duration of all activities with energy expenditure above 3 METs. The *PPGIS mobility log* is synced with the CSV data timeline to remove sleep time and to analyze domain-specific results. Any days with less than 10 h of wear-time, and any measurements with less than 4 valid days and/or those with zero weekend days, will be excluded from the analysis [47]. The final results are normalized to 16 h waking time and weighted between weekdays (weight 5/7) and weekend days (weight 2/7).

*Children’s independent mobility* (CIM). CIM will be operationalized on two levels: as a set of *mobility licenses* parents give to their children (*parent* and *child PPGIS questionnaires*) and as the actual *mobility patterns* of children according to the original CIM survey by Hillman et al. 1990 [40] (*child PPGIS questionnaire*).

*Mobility licenses*. In the *child PPGIS questionnaire* children will be answering questions regarding the mobility license granted to them by their parents [9, 40]. Both children and parents will be asked whether the child is allowed to cross main roads alone, travel home from school alone, and travel on buses alone. Children will also answer if they are allowed to cycle on main roads alone. In the *parent PPGIS questionnaire*, respectively, in addition to the three previously mentioned question, the parents will report if their child can go on their own to places other than school or to go out alone after dark. The mobility license questions will be answered using a dichotomous scale (0 = no, 1 = yes). A mobility license score will be calculated as a sum of aforementioned six items as according to the original study by Hillman and colleagues (1990).

*Mobility patterns*. Children’s actual independent mobility patterns will be studied with *child PPGIS questionnaire*. Children will be asked about their independent mobility to and from school and other destinations on the day they answer the *child PPGIS questionnaire* at school and on a typical day. Dichotomous variables are created from the answers as 1 = travelled without adults or older children during both journeys (travelled “on my own” or “with child/children of same age or younger”) and 0 = travelled with adults/older children (travelled with “parent,” “another adult,” or “older child/teenager”) [9]. A mode value will be created and used to determine children’s actual independent mobility. Second, children will answer questions about their school and other travel modes. Travel mode choice will be surveyed as walking, bicycle, public transit, car, school shuttle bus, skating or scooting or other mode of transport. The school travel mode will be constructed into a dichotomous variable but differing from the original CIM research [33], we will include public transit as an active mode of transportation (walking, bicycle, public transit) and include car and school bus as inactive modes.

*Use of public transit* on a typical week will be assessed based on the travel mode choices children and adults map in the *child* and *adult PPGIS questionnaires*. During the accelerometer measurement week children will map their actual travel mode choices, including use of public transit, in the *PPGIS mobility log*.

*Subjective public transit accessibility* will be assessed in the *child* and *parent PPGIS questionnaire* based on Lättman et al. 2016 [41]. The participants are asked to evaluate their perceived public transit accessibility on a 7-point Likert scale on four different measures; It is easy to do (daily) activities with public transport, If public transport was my only mode of travel, I would be able to continue living the way I want, It is possible to do the activities I prefer with public transport and Access to my preferred activities is satisfying with public transport.

*The experienced implications of free public transit to everyday well-being and equality* will be assessed through guided-tour-interviews and observations, field notes in the ethnography study. *How free bus rides shape the habits and routines* of the children’s and parent’s everyday mobility and lifestyles will be studied with in-depth- interviews with the families in the ethnography study. Finally, *how families argue the necessities or restrictions of CIM* (health and well-being, freedom, security, socio-economic, ecological dimensions) will be studied with in-depth-interviews with the families, as well as through observations and field notes.

### Statistical analysis

Accessibility measures were compared with T-tests with Bonferroni corrections (α_altered_ = .05/12) between schools of Mikkeli and Kouvola to determine if they are comparable to each other and to create comparable school pairs that do not differ from each other statistically significantly. Ten accessibility measures (Table 1) were selected for further analysis for school pairing as they did not show statistically significant differences between the schools. The primary and secondary outcomes will be compared between towns using a hierarchically nested fixed effects ANOVA, where the children will be nested within the cluster factor and compared between the fixed town factor. To study the mechanisms, the models are extended with neighborhood (subjective and objective public transit accessibility), individual (actual use of public transit), as well as domain level factors (public transit use for school commuting and commuting to leisure destinations), respectively. Moreover, we will compare MVPA and sitting time during travel time, school time, non-school time and hobbies, with the same design. The mechanisms will be further studied with structural equation modelling and/or advanced discrete choice modelling (e.g. Integrated choice and latent variable models) in order to study the influence of travel cost (free/not free) on travel mode choice, MVPA and CIM while considering the effect of other latent (e.g. children’s perceptions of safety and security, children’s perception of transit accessibility, parent’s perceptions of safety and security, parents’ perceptions of transit accessibility and of degree of environmental accountability) and objective (e.g. objective accessibility measures) factors.

## Discussion

Integrating physical activity interventions into public policy, like transportation and city planning, is a way to shift physical activity level of societies [48]. The Finnish Basic Education Act guarantees free public transit for long (≥5 km) school journeys. In 2017, city of Mikkeli extended this public service for all children and for the whole day, throughout the year. The purpose of this study is to measure how free public transit affects children’s physical activity time as compared to children living in a reference town without such a service. In addition, children’s independent mobility and sitting time will be studied between the two cities. This study will extend the current evidence base in many ways. We will use novel device-based activity monitoring, which will enable capturing transit-related and non-transit-related MVPA and sitting patterns during the whole day of children. Despite such benefits being reported in adults, the data in children is limited to measured school transit-related physical activity time, or self-reported total walking time [10, 11, 19, 23, 24]. Because the children will wear the activity monitor on their thigh, we will be able to measure time spent standing and cycling, which are inappropriately captured by waist-worn devices, but may be important physical activity modes for both active and public transit users. The interactive mapping technology (PPGIS) is used to capture both children’s and parent’s places of interest as well as their independent and dependent mobility patterns. This detailed mobility data will help us understand how free public transit shapes the families everyday life mobility. Finally, we will use ethnography to get insights on the social and cultural effects and mechanisms of the free public transit in children’s and parent’s everyday life. We believe this mixed methods approach will enable us to identify both the perceived and measured effects at the individual, family, school district, as well as the town levels.

A socio-ecological model of health behavior suggests that interventions should not focus only on intrapersonal factors, but also on the interrelationship between the individuals and their social, physical and policy environment [49, 50]. However, reviews on physical activity interventions in children point out that the majority of evidence is from interventions focusing only to the intrapersonal and social factors, and that their effectiveness is typically small [51, 52]. Moreover, limited evidence exists on the mediating and moderating mechanisms that could enlighten the reasons why the intervention effectiveness remain low [51]. For example, degree of independence [53], autonomy-supportive climate [54], the support of physical environment on walking and cycling [36] or access to facilities [15] are associated with increased physical activity. Research that focuses simply on the individual or social influences on physical activity have been criticized for failing to acknowledge the environment as well as the policy context where the behavior actually takes place [25]. The present setup and a combination of participatory mapping technology with device-measured physical activity enables us to model the effects at different levels of the socio-ecological model, including the policy-level. We will compare device-measured physical activity and CIM at the levels of political action (free vs. non-free public transit), neighborhood (objective public transit accessibility), family (CIM and parental support for physical activity) and individual level (subjective public transit accessibility, actual use of public transit and CIM).

To assist recruitment design, in this paper we report public transit accessibility in different Mikkeli and Kouvola regions. This accessibility data is interesting already on its own. The bus stop density and number of trips decreases considerably in car and out of urban zones, as compared to the urban zone. This accessibility difference may have a significant impact on the public transit use, as well as the potentially ensuing MVPA and CIM benefits, but this has not been fully considered in the previous studies. In addition, we will assess perceived public transit accessibility at children’s’ home environment, which will further enrich the accessibility data from the child’s perspective. We will test these potential mechanisms in the secondary analyses as outlined before.

Independent physical activity improves health at childhood and provides an important foundation for lifelong physical activity and travel habits. This study will provide information about effects of a political action, provision of free public transit for all children, on the children’s physical activity and independent mobility patterns. These results will assist public health, transportation and land use planning policy makers to consider how their decisions will affect children’s physical activity habits.

## Data Availability

The datasets generated and/or analysed during the current study are available in the Traficom repository, http://developer.matka.fi/pages/en/waltti-data.php

## Abbreviations

CIM: Children’s independent mobility
IPAQ: International physical activity questionnaire
LoA: Limits of agreement
MDI: Minimum difference of interest
MVPA: Moderate-to-vigorous physical activity time
PPGIS: Public participation geographic information system
PT: Public transit
